# Development and Validation of a Nursing Care Protocol for Laser Therapy in Pressure Injuries: Methodological Study

**DOI:** 10.3390/ijerph23050541

**Published:** 2026-04-22

**Authors:** Beatrice de Barros Lima, Alessandra Conceição Leite Funchal Camacho, Harlon França de Menezes, Suelem Frian Couto Dias, Richardson Augusto Rosendo da Silva

**Affiliations:** 1 Academic Postgraduate Program in Health Care Sciences, Aurora de Afonso Costa School of Nursing, Fluminense Federal University, Niterói 24020-091, RJ, Brazil; alessandracamacho@id.uff.br (A.C.L.F.C.); harlonmenezes@id.uff.br (H.F.d.M.); suelemfrian@id.uff.br (S.F.C.D.); 2 Postgraduate Program in Nursing, Nursing Department, Federal University of Rio Grande do Norte, Natal 59070-405, RN, Brazil; richardson.augusto@ufrn.br

**Keywords:** pressure ulcer, laser therapy, wound healing, nursing care, clinical protocols, validation study

## Abstract

**Highlights:**

**Public health relevance—How does this work relate to a public health issue?**

- Pressure injuries affect a significant portion of hospitalized patients, particularly the elderly and critically ill, representing a major burden on healthcare systems in terms of costs, complications, and prolonged hospitalizations.
- The absence of validated protocols for emerging technologies such as low-level laser therapy limits safe, standardized care and contributes to clinical variability in wound management.

**Public health significance—Why is this work of significance to public health?**

- This study provides a validated nursing care protocol for low-level laser therapy in stage 1 pressure injuries, with strong content validity (CVI 0.915) and internal consistency (Cronbach’s alpha 0.91), filling a recognized methodological gap in the literature.
- The protocol integrates international patient safety goals and the Nursing Process, reinforcing safe, evidence-based practice applicable across different healthcare settings.

**Public health implications—What are the key implications or messages for practitioners, policy makers and/or researchers in public health?**

- Nurses and healthcare institutions can adopt this protocol to standardize low-level laser therapy practice, reduce clinical variability, and enhance patient safety in pressure injury care.
- Future research should focus on clinical validation of the protocol’s effectiveness and its incorporation into institutional prevention and treatment programs for pressure injuries.

**Abstract:**

Pressure injuries remain highly prevalent in hospital and home settings, particularly among elderly patients, intensive care unit patients, and individuals with impaired mobility, generating significant healthcare and economic impacts. Although low-level laser therapy has been explored as an adjunctive therapy to accelerate healing, few validated protocols exist to guide its systematic application in clinical nursing practice. This methodological study aimed to develop and validate a nursing care protocol for low-level laser therapy in stage 1 pressure injuries, conducted in three stages: integrative literature review, protocol development, and content validation using the Delphi technique with specialist nurses selected via the Lattes Platform. Judges evaluated the protocol using a five-point Likert scale, and validity was assessed by the Content Validity Index (CVI) and Cronbach’s alpha, both with minimum acceptable values of 0.80. The integrative review identified four studies supporting low-level laser therapy efficacy, informing the protocol’s technical parameters. Thirty-one specialists participated in the first Delphi round and 25 in the second, achieving a Global CVI of 0.915 and Cronbach’s alpha of 0.91, with all items reaching consensus. The validated protocol demonstrated satisfactory content validity and internal consistency, supporting its clinical applicability and potential to standardize nursing practice and reinforce patient safety. Although the protocol demonstrated satisfactory methodological validity, further clinical studies are needed to assess feasibility, implementation, and effectiveness in routine nursing care.

## 1. Introduction

Pressure injuries (PI) constitute a chronic condition of high prevalence across diverse healthcare settings, particularly among elderly, critically ill, and mobility-impaired patients. This condition undermines quality of life, increases the risk of infections, prolongs hospital stays, and substantially raises healthcare system costs. The literature reports hospital prevalence rates reaching up to 40%, underscoring both the magnitude of the issue and its significance as a public health challenge [1,2].

The incidence of pressure injuries (PI) remains high in hospital and home settings, especially among patients with multiple comorbidities. Pressure injuries are classified into stages according to tissue involvement. Stage 1 pressure injury is characterized by intact skin with non-blanchable erythema over a localized area, which may be preceded by changes in temperature, consistency, or sensation. Early identification at this stage is critical, as prompt interventions may prevent progression to deeper tissue damage, infection, and prolonged treatment. Recent guidelines highlight that PI are more frequent in the elderly, intensive care unit (ICU) patients, and people with impaired mobility, representing a challenge for health systems [2]. In Brazil, reports indicate hospital prevalences between 22% and 40%, highlighting the magnitude of the problem in national contexts [3]. In addition to their high frequency, PI have significant healthcare and economic impacts, including higher rates of infection, pain, prolonged hospitalizations, and high costs [4].

Nursing plays a pivotal role both in the prevention and in the therapeutic management of pressure injuries, employing classical strategies such as repositioning, skin hydration, and systematic risk assessment. However, given the increasing complexity of cases and the demand for more effective outcomes, there has been a progressive incorporation of innovative technologies into care practices, among which low-level laser therapy (LLLT) stands out. This technology has shown potential to accelerate wound healing and modulate inflammatory processes, yet its application still lacks validated protocols that ensure safety, standardization, and reproducibility [4]. LLLT is a photobiomodulation technology that uses low-intensity light, typically in the red or infrared spectrum, to stimulate cellular metabolism, increase ATP production, modulate inflammatory mediators, improve local microcirculation, and promote collagen synthesis. In pressure injury care, LLLT is applied directly over or around the lesion in a punctual or scanning technique, depending on the clinical objective and wound characteristics. Its use has shown potential to reduce inflammation, relieve pain, and accelerate tissue repair [5].

The absence of effective and validated protocols compromises not only the quality of care but also patient safety and the efficiency of healthcare services [5,6]. Although prevention protocols for pressure injuries, including repositioning, skin hydration, nutritional support, and systematic risk assessment, are well established and supported by robust evidence, there is still a lack of validated nursing protocols specifically designed to guide the safe and standardized application of low-level laser therapy in pressure injury treatment. This methodological gap limits reproducibility, compromises patient safety, and hinders the incorporation of this technology into clinical nursing practice. Several national studies reinforce the effectiveness of prevention protocols [7,8,9]. In a study conducted in an ICU, implementing the protocol led to a significant increase in preventive actions by the nursing team, including risk assessment, skin hydration, and patient repositioning, demonstrating the relevance of standardized procedures [10]. This evidence underscores the central role of nursing in reducing the occurrence of PI.

In the field of technological innovation, low-level laser therapy (LLLT) has been explored as an adjunctive therapy to accelerate the healing of PI [11]. Published reports have described significant improvement in PI with the application of LLLT, as assessed by the Pressure Ulcer Scale for Healing and Nursing Outcomes Classification [12], highlighting the accuracy and depth of nursing assessment, also including theoretical models in the field [13]. These results suggest promising clinical potential but reveal the need for structured and validated protocols.

The literature indicates that although studies demonstrate the benefits of LLLT, few have validated protocols to guide its systematic application in clinical practice. The methodological gap prevents reproducibility of results and integration of the technology into nursing care protocols [14]. Well-designed protocols are essential to ensure the safe and effective application of the technology.

In this scenario, the development and validation of robust care protocols become essential. Supported by rigorous methodologies, such instruments enable the standardization of care, reduce clinical variability, and foster improved patient outcomes. The literature highlights their relevance in the prevention and management of pressure injuries, particularly in high-complexity contexts and in services with limited resources. Thus, the creation of specific protocols to guide the use of this technology proves indispensable, as it strengthens professional autonomy, enhances the quality of nursing practice, and broadens the visibility of nursing as a leading actor in the care of chronic conditions such as pressure injuries.

Given the above, this study aimed to develop and validate a nursing care protocol for the application of LLLT to treat PI.

## 2. Materials and Methods

### 2.1. Study Design

This methodological study was carried out between August and November 2025. It was divided into the following stages, which were carried out at different times: (1) review of the scientific literature on laser therapy protocols for PI; (2) development of the first version of the protocol; and (3) validation of the protocol with specialist nurses.

### 2.2. Stage 1: Integrative Literature Review

In the first stage, an integrative literature review was conducted to survey previously published studies on LLLT care protocols for PI. The integrative review followed six methodological steps: identification of the research question, literature search, study selection, data extraction, critical appraisal, and synthesis of findings. This review was based on the PICO strategy: (P: Population/patients; I: Intervention; C: Comparison/control; O: Outcome), where P: adults and elderly people with PI; I: application of care protocols for the use of LLLT; C: conventional care without a care protocol or without the use of laser therapy; O: improvement in healing and reduction in PI treatment time.

The guiding question was “What evidence is available regarding nursing care protocols for the use of low-level laser therapy in stage 1 pressure injuries?” The search strategy combined controlled descriptors and keywords related to pressure injury, low-level laser therapy, wound healing, and nursing care, using Boolean operators (AND/OR) adapted for each database.

Inclusion criteria were: original studies published in Portuguese, English, or Spanish; studies involving adult or older adult patients with pressure injuries; studies reporting technical parameters or nursing-related care recommendations for low-level laser therapy. Exclusion criteria were: review studies, editorials, dissertations, duplicated records, studies unrelated to pressure injuries, and studies lacking sufficient technical detail.

The search took place between May and July 2025 in the following recognized health databases: Cumulative Index to Nursing and Allied Health Literature (CINAHL); Latin American and Caribbean Health Sciences Literature (LILACS); Scopus; and Medical Literature Analysis and Retrieval System Online (MEDLINE), via PubMed. The Health Sciences Descriptors (in Portuguese, Descritores em Ciências da Saúde—DeCS) and Medical Subject Headings (MeSH) were used, including “Pressure Ulcer” and “Laser Therapy”, along with their synonyms, according to the databases.

The integrative review initially identified 148 studies across the selected databases. After removal of duplicates and title/abstract screening, 19 full-text articles were assessed for eligibility. Of these, four national studies met the inclusion criteria and were included in the final synthesis. These studies specifically addressed low-level laser therapy in pressure injury care and provided technical parameters relevant to protocol development.

Although only four studies met the eligibility criteria, all were critically appraised and considered relevant due to their direct applicability to pressure injury care and laser therapy technical parameters. The protocol was not based solely on these studies, but also on international pressure injury guidelines [2], patient safety recommendations [15], nursing legal frameworks particularly the Brazilian Federal Nursing Council Resolution No. 736/2024 [16], and expert consensus [17], which strengthened its methodological robustness.

### 2.3. Stage 2: SOP Development

In the second stage, the first version of the Standard Operating Protocol (SOP) was developed based on the synthesis of evidence identified in the integrative review and on recommendations from the Guide for the Construction of Nursing Care Protocols. This stage involved defining the protocol structure, technical laser parameters, patient safety recommendations, inclusion of pre- and post-procedure care, documentation standards, and alignment with the Nursing Process [18]. It was structured into the following items: objective, scope, description/definitions, standard protocol, pre-procedure guidance for patients/family members, description of materials, detailed description of activities, critical points/risks, post-procedure care, records, and references.

### 2.4. Stage 3: Content Validation (Delphi)

For the third stage, content validation [19], the Delphi technique was used, with access to specialist nurses regarding the protocol proposal, requiring at least 2 rounds to reach consensus. To this end, nurses registered on the Lattes Platform, maintained by the Brazilian National Council for Scientific and Technological Development (in Portuguese, Conselho Nacional de Desenvolvimento Científico e Tecnológico—CNPq), were selected. The search term “Laser Therapy” was used. In addition, the following filters were applied on the homepage: Academic Background/Qualification: All; Country: Brazil; Region/State: All; and Professional Activity: Broad Area: Health Sciences; Area: Nursing; Subarea: Adult and Elderly Health Nursing; Specialty: All.

In addition to the Lattes search, professionals who were authors of scientific publications on laser therapy and met the inclusion criteria were invited to participate. When specialists from the research universe were available, the “snowball” sampling technique was also adopted, in which participants referred others. The category ‘Others’ refers to professionals holding lato sensu postgraduate certifications or advanced professional qualifications not strictly classified as academic degrees but meeting at least two eligibility criteria (e.g., clinical experience, publications, or participation in research projects).

The inclusion criteria adopted were: thesis/dissertation/specialization in the area of interest, participation in research projects in the area of interest, professional practice, published works, experience in the field of instrument validation or educational materials. To define the sample size of experts, the statistical formula $n=Z_{1-\alpha /2}^{2}\cdot p\cdot (1-p)/e^{2}$ was used, where $Z_{1-\alpha /2}$ corresponds to the confidence level adopted, p to the expected proportion of agreement among experts, and *e* to the acceptable sampling error. A confidence level of 95%, an expected proportion of 85%, and a sampling error of 15% were considered, resulting in an estimated minimum sample of 22 experts [20].

A total of 144 nurses identified through the Lattes Platform were initially invited by email. To enhance sample adequacy and minimize recruitment losses, an additional 17 specialists identified through publication records and snowball sampling were also invited. In the first Delphi round, 31 specialists agreed to participate and completed the evaluation instrument. In the second round, 25 specialists completed the reassessment; six participants were lost to follow-up due to non-response within the predefined period.

Experts were considered eligible if they met at least two of the following criteria: postgraduate specialization, master’s degree, or doctoral degree in wound care or related fields; clinical experience in laser therapy or wound management; authorship of scientific publications on laser therapy or pressure injuries; participation in research projects related to wound care; or previous experience in validation studies.

Although three participants reported no direct experience with laser therapy, they had extensive expertise in wound care, clinical nursing, or protocol validation, which are essential competencies for content validation studies.

Recognizing the recurring difficulties in recruiting experts for instrument validation studies, it was decided to increase the number of invitations sent to ensure the minimum recommended number was reached. The recruitment process was carried out via email, with invitations to the Free and Informed Consent Form (in Portuguese, Termo de Consentimento Livre e Esclarecido—TCLE) and the research instruments sent, and the research instruments were made available on a digital platform (Google Forms).

Participants received a sociodemographic and professional background form, as well as a validation instrument with protocol items. The validation form used a five-point Likert scale from (1) strongly agree to (5) strongly disagree. After each item set, an open field allowed for comments, suggestions, corrections, or proposed changes.

The experts had 90 consecutive days from receipt of the material to complete and return the forms. It should be noted that the steps were conducted by the principal investigator, with support from a team of researchers experienced in the subject matter and the adopted methodology.

After the second round of evaluation was completed, the protocol was reformulated, incorporating the suggestions considered relevant. The revised version was again submitted to the experts for final review and confirmation of the changes made. All participants agreed with the content presented and endorsed the final version of the instrument for application.

A detailed characterization of Delphi participants was added in Table 1 to improve transparency regarding the profile and expertise of the specialist judges.

The apparent inconsistency between the variables ‘experience with laser therapy’ and ‘length of experience with laser therapy’ is explained by differences in response completion. While three participants explicitly reported no experience with laser therapy, two additional participants did not provide complete information regarding the duration of experience, being therefore categorized as ‘no experience’ in this specific variable. This discrepancy reflects missing data rather than inconsistency in the dataset and does not affect the overall validity of the sample characterization.

Although the total number of participants was 31, one participant did not complete the variable related to ‘length of experience with laser therapy’, resulting in a total of 30 valid responses for this specific item. Missing data were handled by considering only valid responses for each variable, without compromising the overall analysis.

### 2.5. Statistical Analysis

For analysis, the judges’ responses were entered into Microsoft Excel 2020 and Statistical Package for the Social Sciences (SPSS) version 22.0, and the scores assigned to each item were examined. The relevance of the items was assessed using the Content Validity Index (CVI), and items with a CVI greater than 0.80 (i.e., agreement greater than 80% among the judges) were considered valid. To assess the protocol’s internal consistency, Cronbach’s alpha was calculated, with an ideal value of 0.8 or higher [21].

### 2.6. Ethical Considerations

The study was approved by the Research Ethics Committee of the Faculty of Medicine of the Federal Fluminense University, under Protocol No. 7.807.925 and Certificate of Ethical Review No. 81923524.5.0000.5243.

## 3. Results

### 3.1. Integrative Literature Review

The integrative literature review enabled the identification and critical analysis of four national studies that examined the use of LLLT for the treatment of skin lesions, particularly in the context of early-stage PI. Methodological heterogeneity was observed among the studies, especially with regard to the technical parameters employed, such as wavelength (ranging from 630 to 660 nm), energy density (between 3 and 6 J/cm^2^), and frequency of application (two to three times per week).

Despite these variations, all studies converged on positive clinical outcomes, including accelerated healing, reduced local inflammation, and improved tissue conditions, reinforcing the effectiveness of the technology when used judiciously and systematically. Furthermore, the findings highlighted the need to incorporate the procedure into the Nursing Process, with continuous clinical evaluation, informed decision-making, and systematic documentation of the interventions performed.

To enhance transparency and provide detailed evidence supporting protocol development, Table 2 presents the characteristics of the studies included in the integrative review, including their aims, methods, main findings, and specific contributions to the construction of the protocol.

### 3.2. Standard Operating Protocol Development

Based on the synthesis of this evidence, it was possible to structure the Standard Operating Protocol (SOP) for laser therapy aimed at treating stage 1 PI, covering the stages of care in an organized manner, from the indication and contraindication of the method to a detailed description of the materials, application technique, and pre- and post-procedure care. The integrative review directly supported the definition of the technical parameters adopted in the protocol and the standardization of care procedures, thereby reducing variability in clinical practice and promoting greater safety in the use of the technology. The SOP was constructed to ensure consistency between the available scientific evidence and everyday care practices, ensuring applicability in the institutional context and alignment with nursing’s legal and technical competencies.

In addition, the review’s results guided the explicit incorporation of international patient safety goals into the protocol, with an emphasis on Goals 1 (correct patient identification) and 5 (hand hygiene). These goals were operationalized in the SOP through the requirement of systematic verification of patient identification before performing the procedure, signage in the environment during laser therapy application, and standardization of hand hygiene steps before and after the intervention.

### 3.3. Content Validation

After the protocol was developed, the content was validated with the participation of specialist nurses. Regarding the specialist nurses’ assessments of the protocol items, a Global CVI of 0.915 and a Cronbach’s alpha of 0.91 for the entire instrument were obtained. All nurses noted that the protocol was comprehensive and served as an initial measure for this action in health institutions.

To improve readability and facilitate practical use, the final validated protocol was reorganized into concise operational domains in Table 3, while detailed technical instructions and procedural specifications were recorded in subitems. This approach preserves transparency regarding validation metrics while improving clarity for readers and clinicians.

The first version of the instrument sent to the specialists was subdivided into individual items. However, the format was revised and accepted for subdivision after the first round to clarify the evaluation and better delineate the protocol’s stages. Thus, the items were dissolved into sub-items and evaluated in the second round.

Other suggestions were accepted in the first round, including removing the dose calculation, since evidence shows there is no need to calculate dose per area in LLLT, and removing the prescribed technical scan [26].

The first version of the protocol consisted of 12 main items organized in a broad format. After the first Delphi round, suggestions from the experts led to the subdivision of these items into operational sub-items to improve clarity, applicability, and evaluation precision. No new thematic domains were added; rather, the content was reorganized to enhance comprehensibility and facilitate practical implementation.

Following expert recommendations, technical instructions related to scan area calculation and scanning techniques were removed from the final protocol to improve clarity and reproducibility. The validated final version exclusively recommends punctual application with standardized parameters for stage 1 pressure injuries.

## 4. Discussion

The characteristics of the participating specialist nurses significantly qualified the analysis of the protocol, as they are professionals with consolidated careers, advanced practice, and technical-scientific expertise in wound care. This profile reflects the progression of professional specialization and investment in specific courses, training, and qualifications, especially in view of the incorporation of emerging technologies in care, which do not replace but complement classic and evidence-based nursing interventions.

In this sense, recent reports reinforce nursing as a visible reference model in wound care, in which new technologies act as transformative elements of care practice, promoting the translation of scientific knowledge into clinical practice and generating a concrete impact on people’s quality of life [11,27].

However, while specialists’ expertise strengthened the protocol validation process, the study also highlights structural weaknesses in specialized nursing training. As studies have noted, many specialization courses still have limited content and are insufficiently aligned with the complex demands of the population, the different stages of the life cycle, and the diversity of healthcare settings [28].

Thus, the high qualification of the participating nurses not only legitimizes the results obtained but also reinforces the need for evidence-based care instruments, such as protocols, that function as pedagogical and clinical devices capable of reducing training gaps, supporting decision-making, and sustaining innovative care practices in a safe, standardized, and scientifically based manner.

With regard to the assessments, the items that received CVIs below 1.0 in the first round of Delphi received more suggestions and adjustments, indicating that the greater the technical complexity, the greater the need for conceptual and operational refinement. In the item “Definition” (CVI 0.87), the diagnostic difficulty of stage 1 was reinforced, especially in dark skin, since the literature points to the delay of health professionals in detecting the first signs of PI, such as changes in skin color [4,29].

The items “Indications” (CVI 0.89), “Contraindications” (CVI 0.88), and “Standard protocol” (CVI 0.90) also required greater consensus, as they involved conceptual alignment with international guidelines and direct risk of diagnostic error, with an impact on patient safety.

The need to review these items reflects the clinical complexity of assessing stage 1 PI and the importance of clear criteria to avoid inappropriate or delayed interventions. Thus, a valid and consistent protocol is necessary, highlighting the importance of a specialized approach to optimize clinical outcomes [30].

The review of technical parameters highlighted the need to standardize care, reduce empirical practice, and strengthen the safe use of technology in nursing. The literature presents significant heterogeneity in parameters, requiring greater standardization in methods regarding ideal treatment parameters, such as the choice of the most appropriate wavelength and the ideal energy dosage [30]. In this sense, the Delphi method served as a device for harmonizing clinical practice, since it was able to methodologically support the opinion and formalization of a protocol such as the one presented, strengthening guidelines and specificities.

The limited number of eligible studies and the heterogeneity of technical parameters identified in the literature require cautious interpretation of the protocol’s technical recommendations. Variations in wavelength, energy density, and frequency of application across studies reflect the lack of robust standardization in the field. Therefore, the protocol should be understood as an evidence-informed and expert-refined instrument, rather than a definitive clinical standard. This reinforces the importance of future clinical validation studies.

The item “Description of activities and patient safety” (CVI 0.81) was the most powerful topic of discussion. This indicated greater concern among specialists with risk, flow, and safety. There was strong alignment with the International Patient Safety Goals, especially Goals 1 and 5 or 6, reaffirming the commitment to safety for all and to quality of care [31].

Continuing with the item described above, a score was assigned for the use of laser therapy to prevent PI. It should be noted that, although this therapy is considered promising in some cases, widely recognized guidelines, such as those of the National Pressure Ulcer Advisory Panel (NPUAP) and the Brazilian Society of Dermatology Nursing (SOBED), emphasize as priority measures changing position every two hours, proper skin care, the importance of adequate nutrition, and effective management of excretions, including urine and feces [32,33].

In addition to these points, the same item also suggests using the spot technique, employing red and infrared light, since this approach allows greater control and assurance of the dosimetry applied to the area to be treated with LLLT. In contrast, the scanning technique does not allow for precise control or exact localization of light distribution in the tissue.

The items “Critical points,” “Risks,” and “Post-procedure care” (CVI 0.89–0.90) reflect concerns about biosafety. Compliance with the recommendations for use of the device and the safety standards established by the competent authorities is noteworthy [34]. The general agreement after the second round of review reinforces that care protocols are not limited to technique, but incorporate ethical, legal, and professional safety dimensions. Technological advances can act as a facilitating element in meeting patient safety goals by encouraging individual participation in care and providing tools that improve the practice of healthcare professionals [35]. Furthermore, this result reinforces the methodological role adopted here as an effective strategy for qualifying clinical instruments, especially when they involve emerging technologies and require integration into the Nursing Process.

The construction and validation of the proposed care protocol reaffirm the centrality of the Nursing Process as the structuring framework for safe, high-quality care for people with PI. The assessment stage, supported by the clinical classification of the injury and analysis of tissue conditions, underpins the nursing diagnosis, especially the risk and presence of PI, guiding coherent clinical decisions. Care planning that incorporates laser therapy as a complementary therapeutic technology highlights nursing’s ability to select evidence-based, individualized, and context-specific interventions. In implementation, the technical description of the procedure, associated with patient safety goals, ensures standardization, reproducibility, and risk reduction. Finally, continuous evaluation, supported by systematic records, consolidates the traceability of care, the measurement of clinical results, and the improvement of professional practice, in accordance with Brazilian Federal Nursing Council (COFEN) Resolution No. 736/2024 [16].

By integrating light-hard technology into the Nursing Process, the protocol strengthens nurses’ clinical autonomy, increases the visibility of their work, and promotes the translation of scientific knowledge into healthcare practice. When systematically incorporated, laser therapy ceases to be an isolated resource and becomes part of intentional, critical, and safe care, aligned with the patient’s needs and the profession’s ethical and legal responsibilities.

Thus, more than standardizing a procedure, this study reaffirms nursing as a protagonist in the incorporation of innovative, evidence-based technologies capable of transforming clinical outcomes and improving care. Protocols based on the Nursing Process not only organize professional practice but also translate science into care, safety into practice, and knowledge into real impact on people’s lives.

Although the protocol demonstrated satisfactory content validity and internal consistency, this study did not evaluate clinical effectiveness, feasibility in routine care, or superiority over existing therapeutic approaches. Therefore, the present findings support the protocol’s methodological validity as a structured nursing care instrument, but not its direct impact on healing outcomes. Future studies should assess usability, implementation barriers, and clinical effectiveness in real-world settings.

The main limitations of the study were the reduced availability of indexed research in high-impact journals that specifically addressed the use of LLLT in stage 1 PI, as well as the scarcity of studies that described and quantified laser technical parameters, such as wavelength, energy density, and frequency of application, in a standardized manner. This gap in the literature made it difficult to directly compare findings and reinforces the need for more scientific production on the topic, especially with more robust methodological designs and greater technical detail. Added to this is the slowness in the experts’ responses to the invitation to participate in the validation process, which required extending the period allocated for protocol evaluation, a situation often reported in methodological studies involving expert judges.

Despite these limitations, the study represents a significant advance by proposing and structuring a protocol based on scientific evidence and aligned with the Nursing Process, contributing to the qualification of care practice and the reduction in clinical variability in the use of laser therapy. Also noteworthy is the high agreement among specialist nurses regarding the protocol’s relevance, clarity, and applicability, reflecting the instrument’s alignment with everyday clinical practice and the real needs of patient care for PI.

This consensus highlights professionals’ recognition of the importance of care protocols as strategic tools for patient safety, standardization of care, and the strengthening of nursing’s technical and scientific autonomy.

## 5. Conclusions

The proposed nursing care protocol for low-level laser therapy in stage 1 pressure injuries demonstrated satisfactory content validity and internal consistency, representing a relevant methodological contribution to nursing practice. The protocol offers structured guidance for safe and standardized care; however, its feasibility, implementation, and clinical effectiveness should be evaluated in future studies before routine incorporation into clinical practice. The instrument consists of steps ranging from careful assessment of the injury and the patient’s clinical conditions to defining technical laser parameters, standardizing procedure execution, and systematically recording and continuously reassessing results, ensuring traceability and monitoring of the care provided.

As an implication for clinical practice, the protocol has the potential to support nurses’ work in different care settings, promoting greater safety in the indication and execution of laser therapy, as well as favoring the standardization of care and the incorporation of soft–hard technologies into the routine of health services. Furthermore, its clinical applicability opens the door to future evaluations of effectiveness, impact on care outcomes, and incorporation into institutional programs for the prevention and treatment of PI, thereby strengthening evidence-based practice and nursing care management.

## Figures and Tables

**Table 1:** Characteristics of expert nurse judges (*n* = 31).

Variable	Category	*n* (%)
Sex	Female	23 (74.2)
	Male	8 (25.8)
Age (years)	32–42	16 (51.6)
	43–53	13 (41.9)
	≥54	2 (6.5)
Years of professional experience	1–10	5 (16.1)
	11–20	12 (38.7)
	≥21	14 (45.2)
Academic degree	Specialization	15 (48.4)
	Master’s degree	5 (16.1)
	Doctorate	7 (22.6)
	Others	4 (12.9)
Area of practice	Clinical care	6 (19.4)
	Teaching/Education	3 (9.7)
	Management	3 (9.7)
	Clinical specialties *	19 (61.2)
Professional experience **	Clinical practice	28 (90.3)
	Teaching	7 (22.6)
	Research	2 (6.5)
Experience with laser therapy	Yes	28 (90.3)
	No	3 (9.7)
Length of experience with laser therapy	≤5 years	20 (64.5)
	>5 years	5 (16.1)
	No experience	5 (16.1)
Training in laser therapy	Attends courses/conferences	30 (96.8)
	Does not attend	1 (3.2)

* Includes cardiology, dermatology, stomatherapy, aesthetics, and other specialties. ** Multiple response variable.

**Table 2:** Characteristics of included studies on LLLT in pressure injury.

Study	Aim	Methods	Main Findings	Contribution to Protocol Development
**Graneiro et al., 2023** [22]	To develop a prototype of a low-level laser therapy protocol for the treatment of pressure injuries and diabetic ulcers	Integrative review	The study structured care into pre-procedure, intra-procedure, and post-procedure actions, emphasizing standardization of clinical conduct and safety measures	Supported the structuring of procedural steps and organization of care (before, during, and after intervention), contributing to protocol standardization and safety
**Sousa et al., 2022** [23]	To report the experience of a specialist nurse in managing a clinical case of an elderly patient with stage 3 pressure injury treated with low-level laser therapy	Experience report (case study)	Application of red (660 nm) and infrared (808 nm) wavelengths, with 1 J/cm^2^ distributed across lesion borders and center, resulting in improvement in tissue conditions and healing progression	Reinforced clinical applicability and supported definition of wavelength and energy density parameters used in the protocol
**Blascovich et al., 2022** [24]	To describe parameters and protocols used for low-level laser therapy in wounds of diabetic patients	Integrative review	Use of wavelengths below 700 nm (red light), power density around 30 mW, and application frequency of 2–3 times per week, with a minimum of ten sessions	Supported standardization of technical parameters, including wavelength, frequency, and number of sessions adopted in the protocol
**Cunha et al., 2017** [25]	To construct and validate an algorithm for laser therapy in wound care	Methodological study based on integrative review	High internal consistency (Cronbach’s alpha = 0.962), demonstrating reliability and methodological robustness of the proposed instrument	Supported methodological structuring and validation approach of the protocol, reinforcing reliability and reproducibility

**Table 3:** Items and sub-items of the final version of the protocol.

Item	Subitem	Delphi 1	Delphi 2
1—Objective	To promote healing of stage 1 PI by applying controlled, localized red light.	1.0	1.0
2—Scope	Qualified nurses.	1.0	1.0
3—Definitions	Intact skin with erythema that does not blanch.	0.87	1.0
	Intact skin with localized area of erythema that does not blanch and may appear different on dark skin.		
	The presence of blanching erythema or changes in sensation, temperature, or consistency (hardening) may precede visual changes.		
	Color changes do not include purple or brown discoloration, which may indicate deep tissue damage.		
4—Indications	Low-level laser therapy may be used as an adjunctive nursing intervention in stage 1 pressure injuries characterized by intact skin with non-blanchable erythema, aiming to modulate inflammation, improve local microcirculation, and support early tissue recovery.	0.89	1.0
	For this protocol, red light at 660 nm with an energy density between 1 and 2 J/cm^2^ is recommended, applied twice weekly according to clinical assessment.		
5—Contraindications	-On the pregnant uterus.	0.88	1.0
	-Neoplasia in the region to be irradiated.		
	-Undiagnosed clinical lesions.		
	-On the skin of patients who use photosensitive substances topically (e.g., isotretinoin, retinoic acid, among others).		
	-Do not apply on tattoos or micropigmentation.		
6—Standard protocol	Standard technical parameters for stage 1 pressure injury care include the use of red light (660 nm), punctual application at a 90° angle, maintaining approximately 1 cm distance from the skin surface, with energy density between 1 and 2 J/cm^2^ per point.	0.90	1.0
	The protocol is exclusively intended for superficial skin involvement and does not apply to deeper injuries, burns, postoperative wounds, or mucosal lesions.		
7—Pre-procedure guidance for the patient/family	Inform the patient/family about the procedure to be performed according to their level of understanding.	1.0	1.0
	Sign the Free and Informed Consent Form for LLLT.		
8—Description of Materials	-Procedure gloves.	0.89	1.0
	-Surgical mask.		
	-Protective film for the device.		
	-02 Protective glasses (black)—ONLY those supplied by the manufacturer.		
	-Laser therapy equipment.		
9—Detailed descriptions of activities	Confirm patient identification according to institutional safety protocol.	0.81	1.0
	Place laser therapy warning signage at the room entrance.		
	Minimize unnecessary circulation in the room during the procedure.		
	Perform hand hygiene before the procedure.		
	Position the patient comfortably to allow clear visualization of the lesion.		
	Ensure protective eyewear is used by the patient and all professionals present.		
	Prepare the equipment according to manufacturer recommendations.		
	Apply the laser perpendicularly (90° angle) to the affected skin area, maintaining approximately 1 cm distance.		
	Use punctual application over the erythematous area according to the predefined energy density.		
	Perform hand hygiene after the procedure.		
10—Critical Points/Risks	Read the manual thoroughly before using the equipment.	0.85	1.0
	Only qualified and properly trained professionals should operate, use, and direct treatments with the equipment.		
	All persons present in the area where laser light is emitted must protect their eyes.		
	Never look directly at the emitted laser light and, above all, do not direct it at anyone other than the person undergoing treatment.		
	Shiny surfaces can reflect laser light toward the eyes.		
	Never irradiate an undiagnosed lesion.		
	Only the components mentioned in the device manual should be used in conjunction with the equipment.		
	The equipment should not be used with a power cord or power supply other than those supplied by the manufacturer, as this may increase emissions or decrease the immunity of the equipment.		
	To avoid the risk of electric shock, the equipment should only be connected to grounded power outlets.		
	Do not connect the power cord to outlets that are difficult to access.		
	When removing the power cord from the outlet, always pull on the plug.		
	No modifications to the equipment are permitted.		
	The air inlets/outlets for equipment ventilation must not be obstructed.		
	Do not apply any type of protective film to the handpiece in order to avoid obstructing the air inlet/outlet for ventilation.		
	Do not install the equipment in a location subject to direct sunlight, excessive dust, or mechanical vibrations.		
	The use of flammable or oxidizable anesthetic gases, such as nitrous oxide (N_2_O) and oxygen, should be avoided.		
11—Post-Procedure Care	The device should be cleaned/disinfected with a soft cloth moistened with 70% alcohol.	0.90	1.0
	Protective glasses can be washed with soap and water or 70% alcohol.		
	Change position every 2 h.		
	Assess the frequency of laser use.		
12—Records	Record the procedure on the Nursing Progress form, documenting the characteristics of the injury, date and time of the procedure, and materials used.	1.0	1.0

PI: Pressure Injury. LLLT: Low-Level Laser Therapy. Values represent CVI per item; CVI ≥ 0.80 was considered acceptable. Source: Research data, 2025.

## Data Availability

The data presented in this study are not publicly available due to privacy and ethical restrictions. Data may be available from the corresponding author upon reasonable request.

## Abbreviations

The following abbreviations are used in this manuscript:

PI	Pressure Injury
LLLT	Low-Level Laser Therapy
SOP	Standard Operating Protocol
ICU	Intensive Care Unit
PICO	Population, Intervention, Comparison, Outcome
DeCS	Health Sciences Descriptors
MeSH	Medical Subject Headings
CINAHL	Cumulative Index to Nursing and Allied Health Literature
LILACS	Latin American and Caribbean Health Sciences Literature
MEDLINE	Medical Literature Analysis and Retrieval System Online
CVI	Content Validity Index
SPSS	Statistical Package for the Social Sciences
TCLE	Free and Informed Consent Form
CNPq	Brazilian National Council for Scientific and Technological Development
NPUAP	National Pressure Ulcer Advisory Panel
SOBED	Brazilian Society of Dermatology Nursing
COFEN	Brazilian Federal Nursing Council
